# Hyaluronan-mediated motility receptor antisense RNA 1 promotes hepatitis B virus-related hepatocellular carcinoma progression by regulating miR-627-3p/High Mobility Group AT-hook 2 axis

**DOI:** 10.1080/21655979.2022.2054151

**Published:** 2022-03-24

**Authors:** Hai Zhuang, Xiaoxia Ma, Xiaoyan Liu, Chao Li, Xinying Li, Ling Wu, Maofei Wen, Wenli Shi, Xiaozhou Yang

**Affiliations:** a Department of Infectious Diseases Ward, Baiyun Hospital Affiliated to Guizhou Medical University, Guiyang, China; b Department of Hepato-Biliary Surgery Ward, Affiliated Hospital of Guizhou Medical University, Guiyang, China; c Department of Infectious Diseases, The Second Affifiliated Hospital of Dalian Medical University, Dalian, China

**Keywords:** HMMR-AS1, HBV-HCC, miR-627-3p, HMGA2

## Abstract

Hepatocellular carcinoma (HCC) is a common malignancy in the world, with high mortality and poor prognosis. Hepatitis B virus (HBV) is one of the key factors implicated in the occurrence of HCC. Increasing evidence suggests that miRNAs play important roles in the development and metastasis of HBV-associated HCC (HBV-HCC). Here, we performed CCK8 (Cell count kit-8), EdU (5-ethynyl-2’-deoxyuridine) incorporation assay, wound-healing assay, transwell assay to study the changes in the cellular phenotype. Luciferase reporter assay, RNA pull-down experiment, RT-qPCR and western blotting were employed to study molecular mechanism. In addition, we also constructed a mouse HCC xenograft model to verify the functional role of HMMR-AS1/miR-627-3p/HMGA2 signal axis *in vivo*. Our study demonstrated that HMMR-AS1 was highly expressed in HCC tissues and cell lines, suggesting its implication in the progression of HCC. In addition, *in vitro* experiments showed that high HMMR-AS1 expression facilitated the migration, invasion, and proliferation of HCC cells. We further revealed that HMMR-AS1 promoted the malignant phenotype of HCC cells by regulating miR-627-3p/HMGA2 axis. Together, our data suggest that HMMR-AS1 regulates HBV-HCC progression via miR-627-3p/HMGA2 axis.

## GRAPHICAL ABSTRACT

**Figure:**
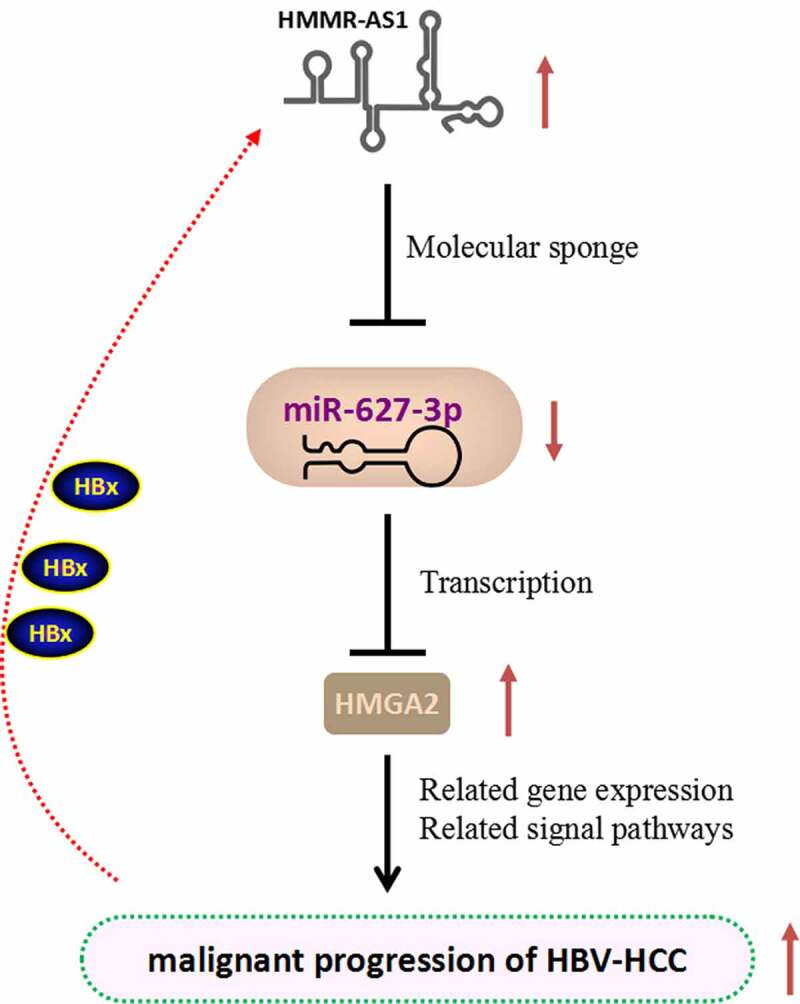

## 1. Introduction

Hepatocellular carcinoma (HCC) is a common malignancy with high mortality and poor prognosis worldwide [1]. The mechanisms involved in the initiation and development of HCC have been under intensive investigation for many years. Risk factors for HCC include chronic hepatitis B and C infection, alcohol addiction, and metabolic liver disease [2,3]. Specially, hepatitis B virus (HBV) is closely related to the occurrence of HCC, and HBV-encoded X protein (HBX) is recognized as an important effector in virus-induced HCC [4]. HBX, a 17 kDa multifunctional protein, regulates a variety of intracellular processes by regulating a variety of cellular targets. It has been reported that HBX expression could promote the invasion and metastasis of HCC [5]. In addition, recent studies have found that HBX can induce the abnormal expression of several microRNAs and affect downstream signaling pathways to promote the development of HBV-associated HCC (HBV-HCC) [^6–9^].

MicroRNAs (miRNAs), a class of non-coding RNAs that play important roles in gene regulation, are implicated in the initiation and progression of many tumors [10]. Recent studies have shown that the dysregulation of miRNAs is associated with tumor progression, including the drug resistance and metastasis [^11–13^]. A previous study reported a total number of 153 up-regulated miRNAs and 206 down-regulated miRNAs in HCC through miRNA gene chip technology, and there are thousands of differentially expressed genes potentially targeted by these miRNAs [14]. As a well-studied miRNA in tumor biology, miR-627-3p plays a regulatory role in the malignant process of osteosarcoma [15], lung cancer [16], colorectal cancer [17] and esophageal cancer [18]. miR-627-3p can mediate the effects of other non-coding RNAs including long non-coding RNAs (LncRNAs) and circRNAs. However, whether miR-627-3p plays a potential role in HBV-HCC is unknown.

LncRNAs are another class of non-coding RNAs that have been extensively investigated in tumor biology. They could regulate the occurrence and progression of tumor frequently through targeting downstream miRNAs [19]. Our previous preliminary experiment found that hyaluronan-mediated motility receptor antisense RNA 1 (HMMR-AS1) was highly expressed in HCC tissues. HMMR-AS1 has been reported to play an important role in lung cancer, ovarian cancer, breast cancer, and glioma [^20–23^]. In lung cancer, HMMR-AS1 has been showed to modulate the sirt6 expression through miR-138, thereby regulating the proliferation and metastasis of lung cancer cells [20]. The potential role of HMMR-AS1 in HBV-HCC has not been studied, and its expression pattern HBV-HCC remains unclear.

In this study, based on previous studies and our preliminary work, we speculated that HMMR-AS1 may affect the expression of the target gene High Mobility Group AT-hook 2 (HMGA2) through miR-627-3p, thereby affecting the progression of HBV-HCC. We analyzed the expression of HMMR-AS1 in HBV-HCC, and investigated its functional roles in HCC cell lines. Through different molecular and functional assays, we further revealed that miR-627-3p/HMGA2 axis mediates the tumor-promoting effect of HMMR-AS1 in HCC.

## 2. Materials and methods

### 2.1. Cell culture

All cell lines used in this study were purchased from ATCC and cultured in a CO_2_ incubator at 37°C. HepG2 and Huh7 cells were cultured in RPMI-1640 medium with 10% FBS. HepG2.2.15 and Huh7-1.3 (stable transfection with HBV genomic DNA) cells were cultured in MEM medium supplemented with 10%FBS and 380UG/ML G418.

### 2.2. Clinical tissues

Tissue samples used in this study were collected from 64 HBV-positive early HCC patients who received hepatectomy at the Second Affiliated Hospital of Dalian Medical University. Among them, 40 cases were male and 24 cases were female (Table 1). All the samples were confirmed with the detection of HBV genomic DNA. Intraoperative resected tumor tissues and paracancerous normal tissues were collected for clinical and pathological verification, and stored at −80°C for subsequent use. The study was approved by the Ethics Committee of The Second Affifiliated Hospital of Dalian Medical University. All participants signed informed consent in this study.

**Table 1.** Collection of basic conditions of HBV-HCC patients

Characteristics	HBV-HCC (n = 64)
Sex (M/F)	40/24
Age (year)	47.23 ± 2.04
HBV DNA (log10 IU/mL)	5.34 ± 0.19
AST (U/L)	87.16 ± 8.42
ALT (U/L)	71.12 ± 12.12
PLT (×10^9^/L)	243.71 ± 17.21

### 2.3. Cell transfection

Recombinant eukaryotic cell expression vector plasmid Prep-HBV 1.2 was preserved in our laboratory. LipofectamineTM 2000 Kit and Opti-Men medium for transfection were purchased from Invitrogen (USA). miR-627-3p mimics, inhibitors, negative controls (NC), and siRNA targeting HMMR-AS1 were all synthesized by GenePharma (China).

Transfections of above molecules into the cells were performed by LipofectamineTM 2000 Kit following the manufacturer’s protocol. 6 µg plasmid, or 200 nM of microRNA mimic or the NC, 100 nM of siRNA were used for transfecting cells in 6 well plate at 80% confluency. To generate cells stably expressing HBV genes, cells transfected with Prep-HBV 1.2 were selected with 500 µg/ml G418 for two weeks, and the cells were maintained in medium with 200 µg/ml G418. For other transfections, cells were harvested 48 h after transfection for further experiments.

### 2.4. Cell prolifetaion assay

CCK-8 kit was purchased from Dojindo Molecular Technologies (Japan) as previously described [5]. Forty-eight hours after transfection, cells were seeded in to a 96-well plate at a density of 1500 cell/well and cultured in a humidified cell culture incubator for 0, 24, 48 and 72 hours. 10 μL of CCK-8 reaction solution was added to the cell culture at indicated time point and incubated for 1 hour. The light absorption value (OD value) in each condition was captured at 450 nm wavelength on a Synergy H1 microplate reader.

### 2.5. EdU assay

EdU reagent (Abbkine, USA) was used to detect the rate of EdU incorporation in DNA synthesis. HCC cell lines were pulse labeled with 50 μM EdU labeling medium for 2 h. Then, cells were fixed with 4% paraformaldehyde (PFA), and permeabilized with 0.5% Triton® X-100 in PBS. After the removal of the solution, 1 × Click-iT® reaction cocktail was prepared based on the manufacturer’s instruction and added to cells for 30 mins incubation. The staining cocktail was removed and cells was washed twice with 100 µL of PBS with 3% BSA. Cells were counter-stained by 500 nM DAPI in PBS and the images were captured under Leica AM6000 microscope.

### 2.6. Real-time quantitative PCR

Total RNAs in cells and tissues were extracted using Trizol reagent (Invitrogen, USA), according to the procedures as previously described [5]. Subsequently, 5 μg of total RNA was used for reverse-transcription into cDNA using RevertAid First-Strand cDNA Synthesis Kit (Thermo Fisher Scientific, USA). The expression of the target genes was detected by the PrimerScript^TM^ RT reagent Kit (Takara, Japan) on a 7500 Real-Time PCR System (Applied Biosystems, CA, USA). β-actin was used as the reference gene and the relative gene expression was calculated by the 2^−ΔΔCt^ method. The sequences of primers in this study are as follows: HMMR-AS1 (forward: 5’-CACAACTCCTGGTTCCTG-3’; reverse: 5’-GGATGGGTATTGGTCTGT-3’); miR-627-3p (forward: 5’-CACTCATCTTTTCTTTG-3’; reverse: 5’-GAGTCTCTTGAGAGTACAT-3’); β-actin (forward: 5’-CCACTGGCATCGTGATGGA-3’; reverse: 5’-CGCTCGGTGAGGATCTTCAT-3’).

### 2.7. Western blotting analysis

Total proteins were extracted from cells and tissues by RIPA lysis and extraction buffer (Thermo Fisher, USA) as previously described [5]. The supernatant containing total protein lysate was quantified by a BCA Protein assay kit (Beyotime, Shanghai, China). 10 ug of total protein was used for SDS-PAGE electrophoresis and then transferred onto the PVDF membrane. After blocking with 5% skimmed milk for 1 hour, the membrane was then incubated with primary antibodies (Cell Signaling Technology, USA) overnight at 4°C. The membrane was washed 3 times with TBST and incubated with HRP-linked secondary antibody (Cell Signaling Technology, USA) at room temperature for 1 hour. Protein bands were developed using an enhanced chemiluminescence kit (Santa Cruz, TX, USA) and photographed on a gel imager system (Bio-Rad, CA, USA). The densitometry analysis was performed with Image J software (Bethesda, MD, USA).

### 2.8. Cell migration and invasion assays

Cell migration was analyzed by wound-healing assays as previously described **[**5**]**. Cells (5 × 10^5^) were seeded into 24-well plates and then a micropipette tip was used to scarp four scratches on each well. Surplus cells were washed away in a serum-free medium. The scratches in the same location were observed at different points under a phase-contrast miscroscope. Cell invasion assay was conducted using transwell coated with Matrigel (Corning, USA). 5 × 10^5^ cells were inoculated into the transwell upper chamber in serum-free medium and 500 μL of 10% serum-containing medium was added to the lower chamber. After 24 hours, culture medium was discarded and cells in the upper chamber were swabbed. Cells in the lower chamber were fixed with 4% PFA and stained with 1% crystal violet. After drying, the cells in the lower chamber (migratory cells) were observed under a microscope.

### 2.9. Dual luciferase reporter assay

Luciferase reporter assay was to proceed as previously described [8]. The wild-type or mutated HMMR-AS1 fragment and HMGA2 3’-UTR fragment were constructed into the PmirGLO luciferase reporter (Promega, USA). The reporter plasmid and Renilla luciferase (hRlucneo) control plasmid were co-transfected into cells with either miR-627-3p mimic or miR-NC in a 12-well plate (1 × 10^5 cells/well) using Lipofectamine 3000 reagent (Invitrogen, USA). Forty-eight-hour post transfection, the relative luciferase activities were measured by Dual-Luciferase Reporter Assay Kit (Promega, USA) on a luminescence microplate reader.

### 2.10. Xenograft mouse model

Thirty male immunodeficient nude mice weighing 30–40 g were randomly divided into two groups (15 mice in each group): (1) NC group (injected with HepG2.2.1 cells infected with sh-NC), (2) sh-HMMR-AS1 (injected with cells infected with sh-HMMR-AS1). 0.5 mL of cell suspension containing 1 × 10^7^ cells was injected into the flank of each mice. Tumor volume were monitored 7, 14, 21, and 28 days post-injection, respectively. Four weeks after tumor cell inoculation, all the mice were euthanized by CO2 asphyxiation, and the xenograft tumors were collected and weighed. The animal experiment was approved by the Ethics Committee of The Second Affifiliated Hospital of Dalian Medical University.

### 2.11. RNA pull-down assay

The bait probe were labeled with biotin via Pierce™ RNA 3’ End Desthiobiotinylation Kit (Thermo Fisher, USA). The harvested cells were lysed and then incubated with biotin-labeled RNA probes. 10% of lysate was saved as input. Then, the molecures associated with probe was pulled down using streptavidin-labeled magnetic beads, and washed 4 times with the lysis buffer. Then the total RNAs in the input and in the pull-down samples were extracted using Trizol reagent, and the relative level of miR-627-3p in the samples was quantified by qRT-PCR.

### 2.12. Statistical analysis

SPSS 19.0 software was used for statistical analysis. The difference between the two groups was analyzed by Student’s t-test, and comparisons among multiple groups were analyzed one-way analysis of variance. The correlation of expression was statistically analyzed by Spearman correlation coefficient analysis. The overall survival of HCC patients was analyzed by Kaplan-Meier survival curve. All experimental data are presented as the mean ± SD of at least three independent experiments. *P* < 0.05 was statistically significant. *P < 0.05, **P < 0.01, ***P < 0.001.

## 3. Results

In this study, we hypothesized that in HBV-HCC, HMMR-AS1 may affect the expression of the target gene HMGA2 through miR-627-3p, thereby affecting the progression of HCC. Our results demonstrated that HMMR-AS1 was upregulated in HCC tissues, which is associated with a poor prognosis in HCC patients. In addition, in vitro experiments showed that high HMMR-AS1 expression facilitated the migration, invasion, and proliferation of HCC cells. We further showed that HMMR-AS1 promoted the malignant phenotype of HCC cells by regulating miR-627-3p/HMGA2 axis.

### 3.1. HMMR-AS1 was upregulated in HBV-HCC tissues and cell lines

**Figure 1.**
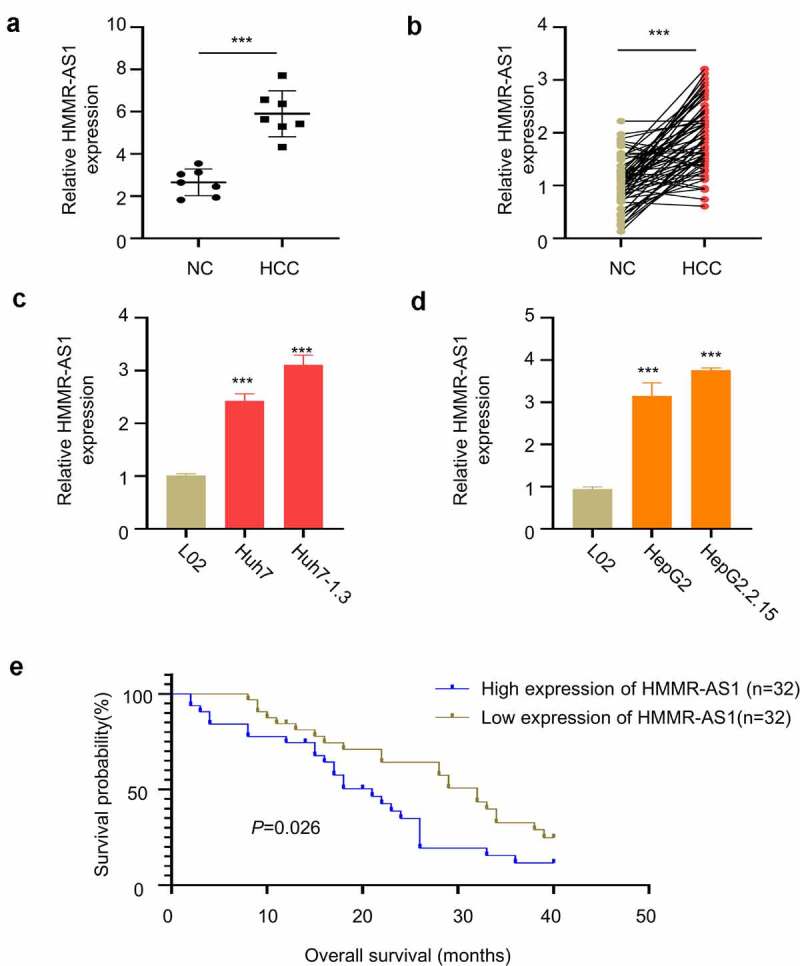
HMMR-AS1 is Highly Expressed in HBV-HCC Tissues and Cell Lines. (a) GSE101728 dataset analysis showed the upregulation of HMMR-AS1 expression in HCC tissues. (b) The expression of HMMR-AS1 in 64 HBV-HCC tumor and paracancerous tissues was detected by qRT-PCR. (c) The expression of HMMR-AS1 in Huh7 cells, Huh7-1.3 (HBV-positive Huh7 cell line), and the normal liver cell lines (L02) was detected by qRT-PCR. (d) HMMR-AS1 expression in HepG2 cells, HepG2.2.15 (HBV-positive HepG2 cell line), and the Normal Liver Cell Lines (L02) was detected by qRT-PCR. (e) The correlation between HMMR-AS1 expression level and overall survival of HBV-HCC patients was assessed by Kaplan-Meier survival curves.

We first obtained GSE101728 microarray data set through GEO database to search for differential expression genes and found that HMMR-AS1 was significantly upregulated in HCC tissues (Figure 1(a)). We then collected 64 pairs of HBV-HCC tumor tissues and paracancerous tissues, and RT-qPCR analysis showed that HMMR-AS1 was highly expressed in HBV-HCC tumor tissues (Figure 1(b)). In addition, the expression of HMMR-AS1 was significantly higher in HCC cell line Huh7 and Huh7-1.3, as compared to normal liver cell line L02 (Figure 1(c)). Moreover, the expression of HMMR-AS1 was further increased in the Huh7-1.3 cell line that stably expressing HBV genome (Figure 1(c)). Similarly, the expression of HMMR-AS1 in HepG2 cells was higher than that of normal liver cell line L02, and its expression level was further increased in HepG2.2.15 cell line (HBV-positive HepG2 cell line) (Figure 1(d)). To further explore the correlation between HMMR-AS1 expression and the patient survival, HBV-HCC patients were divided into HMMR-AS1-high and -low-expression group (n = 32 in each group) based on the median expression value of HMMR-AS1. We found that high HMMR-AS1 expression was associated with a poor prognosis in HBV-HCC patients (Figure 1(e)). Overall, these data suggest that HMMR-AS1 upregulation is associated with the progression of HBV-HCC.

### 3.2. HBX promotes the expression of HMMR-AS1 in HCC cells

**Figure 2.**
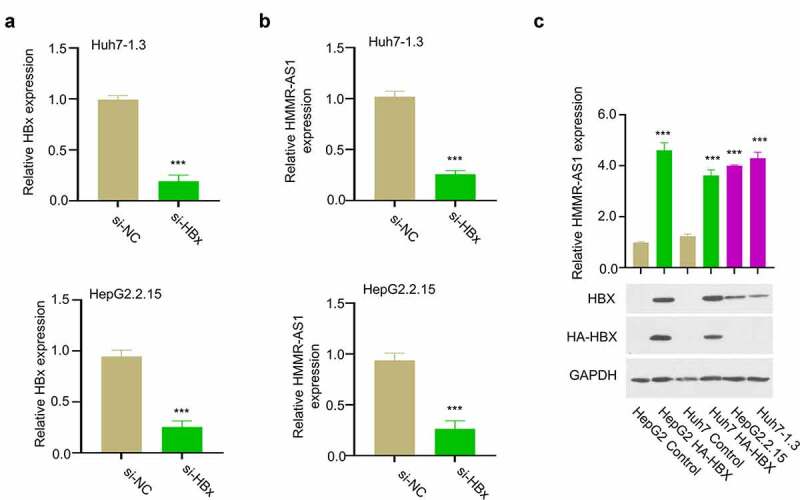
HBX silencing suppressed the expression of HMMR-AS. (a) The knockdown effect of siRNA was verified by qRT-PCR in Huh7-1.3 and HepG2.2.15 cells. (b) HMMR-AS1 expression in different groups of Huh7-1.3 and HepG2.2.15 cells (si-NC, si-HBx) was detected by qRT-PCR. (c) The effects of transient HBX overexpression (HA-HBX) in Huh7 and HepG2 cells and stable HBX expression in HepG2.2.15 and Huh7-1.3 cells (integration of the HBV genome) on the expression level of HMMR-AS1.

HBX is an effector protein encoded by HBV genome, which regulates a variety of intracellular processes by targeting a variety of cellular targets [4]. To investigate the whether HBX is involved in HMMR-AS1 regulation in HBV-HCC, we transfected HBV-positive HCC cells (Huh7-1.3 and HepG2.2.15) with siRNA targeting HBX to reduce HBX level (Figure 2(a)). qRT-PCR analysis showed that the level of HMMR-AS1 expression was downregulated upon HBX knockdown (Figure 2(b)). We also transfected HCC cells with plasmid expressing HA-tagged HBX. The results showed that the transient HBX overexpression or the stable HBX expression in HBV-positive cells both remarkably enhanced HMMR-AS1 expression (Figure 2(c)). Therefore, we conclude that HBX promotes the expression of HMMR-AS1 in HCC cells.

### 3.3 Knockdown of HMMR-AS1 inhibits HBV-HCC cell proliferation, invasion, and migration

**Figure 3.**
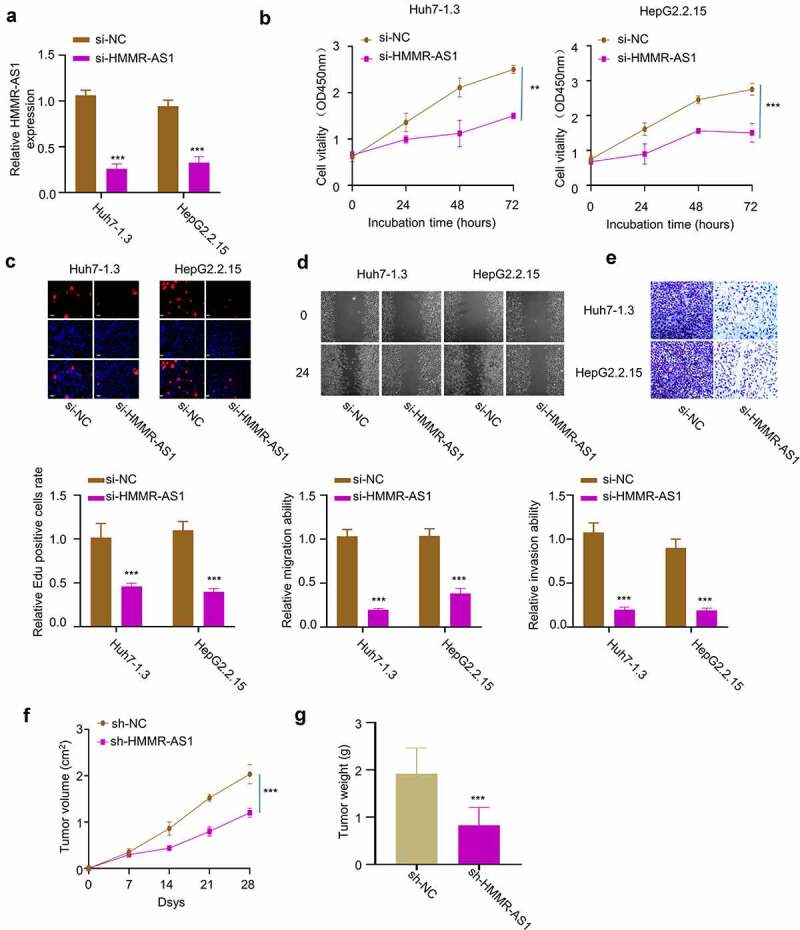
HMMR-AS1 silencing suppresses the proliferation, migration, and invasion of HBV-HCC cells. (a) The expression of HMMR-AS1 in different groups of Huh7-1.3 and HepG2.2.15 cells (si-NC, si-HMMR-AS1) was detected by qRT-PCR. (b) Cell proliferation and EdU incorporate rate (c) in Huh7-1.3 and HepG2.2.15 cells transfected with si-NC or si-HMMR-AS1 were examined by CCK8 and EDU assays. (d) Wound-healing assay was performed to detect cell migration in Huh7-1.3 and HepG2.2.15 cells transfected with si-NC or si-HMMR-AS1. (e) Cell invasion was detected by transwell assays. (f) **and** (g) Subcutaneous tumor sizes and tumor weight in nude mice inoculated with HepG2.2.15 cells transfected with sh-NC or sh-HMMR-AS1.

To investigate the functional role of HMMR-AS1 in HBV-HCC cells, we applied siRNA targeting HMMR-AS1 and demonstrated that it significantly reduced HMMR-AS1 expression in Huh7-1.3 and HepG2.2.15 cells (Figure 3(a)). CCK8 assay demonstrated that HMMR-AS1 knockdown significantly suppressed cell proliferation (Figure 3(b)). EdU incorporation assay further showed that HMMR-AS1 silencing significantly reduced EdU incorporation in DNA synthesis (Figure 3(c)). These data suggest that HMMR-AS1 is required for the proliferation of HCC cells. We next performed wound-healing assay and transwell invasion assay. The results showed that HMMR-AS1 knockdown impaired cell migration and invasion ability in HBV-HCC cells (Figure 3(d,e)). To validate the role of HMMR-AS1 in tumorigenesis, we inoculated HepG2.2.15 transfected with control shRNA (sh-NC) or sh-HMMR-AS1 into nude mice. The subcutaneous tumorigenesis of HepG2.2.15 cells was significantly suppressed after HMMR-AS1 knockdown, as revealed by the reduced tumor volume and weight (Figure 3(f,g)). Taken together, HMMR-AS1 is indispensable for the malignant phenotype of HBV-HCC cells.

### 3.4. HMMR-AS1 interacts with miR-627-3p and regulates its expression

**Figure 4.**
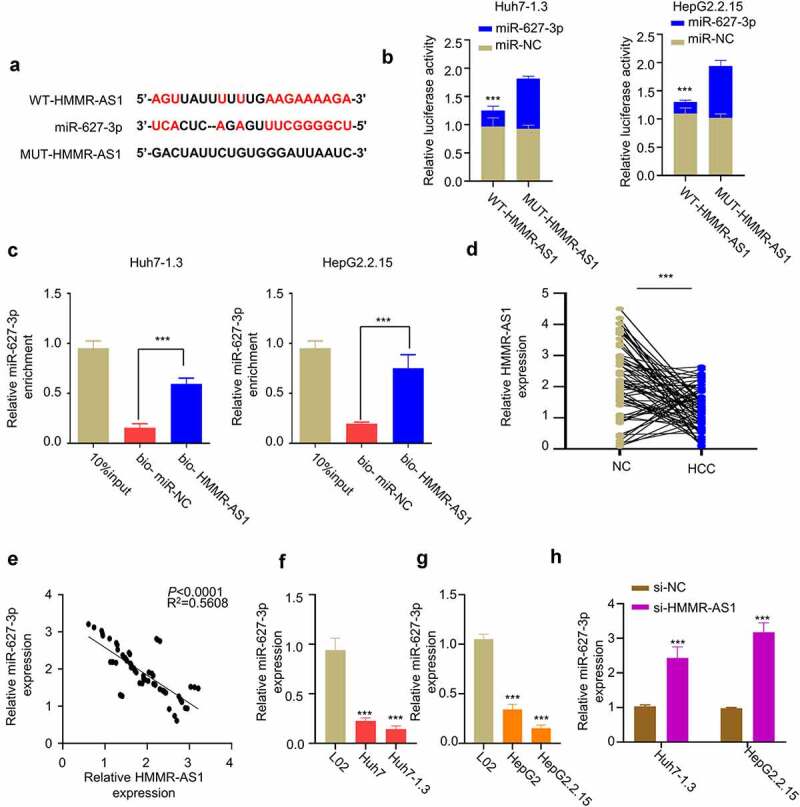
HMMR-AS1 binds to miR-627-3p and modulates its expression. (a) The binding site of HMMR-AS1 and miR-627-3p was predicted by LncBASE analysis. (b) Dual-luciferase reporter assay in Huh7-1.3 and HepG2.2.15 cells transfected with wild-type or mutant HMMR-AS1 luciferase reporter, in the presence of miR-627-3p mimic or miR-NC. (c) RNA pull-down assay using biotin-labeled HMMR-AS1 probe or miR-NC probe. (d) The expression of miR-627-3p in cancerous tissues and adjacent noncancerous tissues (n = 64) was detected by qRT-PCR. (e) Correlation of miR-627-3p and HMMR-AS1 expression in HBV-HCC carcinoma tissues. (f) The expression of miR-627-3p in Huh7 cells, Huh7-1.3 (HBV-positive Huh7 cell line), and the normal liver cell lines (L02) was detected by qRT-PCR. (g) miR-627-3p expression in HepG2 cells, HepG2.2.15 (HBV-positive HepG2 cell line), and the normal liver cell lines (L02) was detected by qRT-PCR. (h) RT-qPCR analysis of miR-627-3p expression in Huh7-1.3 and HepG2.2.15 cells after HMMR-AS1 knockdown.

Through LncBASE online database, we found that HMMR-AS1 contains a binding sequence with miR-627-3p binding sites (Figure 4(a)). We performed dual-luciferase reporter assay in Huh7-1.3 and HepG2.2.15 cells using WT-HMMR-AS1 and MUT-HMMR-AS1 reporters in the presence of miR-NC or miR-627-3p mimic. Compared with miR-NC, miR-627-3p mimic significantly inhibited luciferase activity of WT reporter, and no effect was observed for MUT reporter (Figure 4(b)). Meanwhile, we performed RNA pull-down assay using biotin-labeled HMMR-AS1 probe, which revealed that HMMR-AS1 probe could significantly enrich miR-627-3p in Huh7-1.3 and HepG2.2.15 cells (Figure 4(c)). We then examined how HMMR-AS1 affects the expression of miR-627-3p. First, we found the downregulation of miR-627-3p in HBV-HCC tumor tissues (Figure 4(d)), and the expression of miR-627-3p was negatively correlated with HMMR-AS1 in HBV-HCC tumor tissues (Figure 4(e)). Consistently, miR-627-3p expression in HCC cell lines was lower than that of LO2 cells, which was further reduced in HBV-HCC cell lines (Figure 4(f,g)). When HMMR-AS1 was silenced, miR-627-3p expression level was increased in HBV-HCC cells (Figure 4(g)). Taken together, HMMR-AS1 targets miR-627-3p and negatively regulates its expression.

### 3.5. miR-627-3p negatively regulates the expression of HMGA2 protein

**Figure 5.**
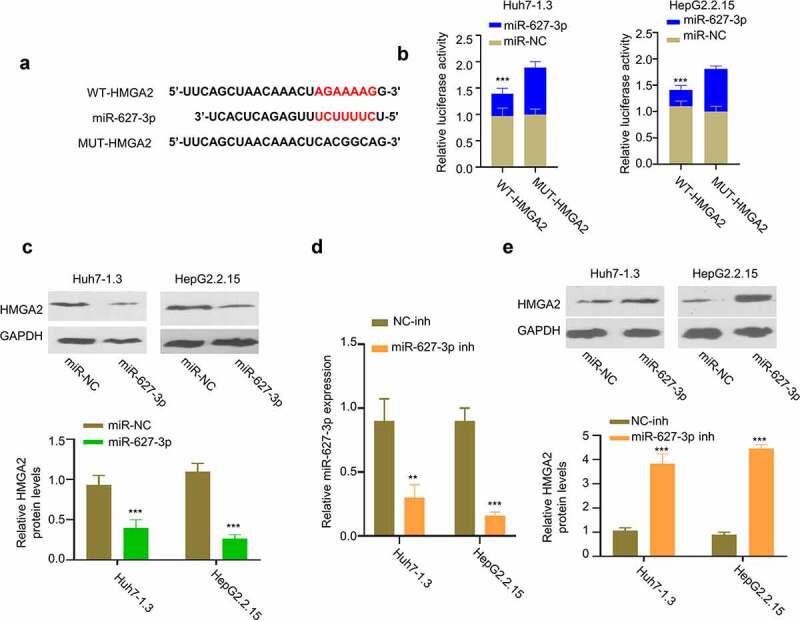
HMGA2 is a target of miR-627-3p. (a) Predicted binding site between HMGA2 mRNA and miR-627-3p was identified by Targetscan online tool. (b) Dual-luciferase reporter assay in Huh7-1.3 and HepG2.2.15 cells transfected with wild-type or mutated HMGA2 luciferase reporter, in the presence of miR-627-3p mimic or miR-NC. (c) HMGA2 protein level in cells transfected with miR-627-3p mimic. (d) The effect of miR-627-3p inhibitor transfection was detected by RT-qPCR. (e) Western blot analysis of HMGA2 protein level in Huh7-1.3 and HepG2.2.15 cells transfected with NC inhibitor or miR-627-3p inhibitor.

We next used TargetScan software to search for target mRNAs of miR-627-3p, and found the presence of miR-627-3p binding sites in the 3’-UTR of HMGA2 mRNA (Figure 5(a)). Dual-luciferase reporter assay showed that miR-627-3p mimic inhibited the activity of WT-HMGA2 reporter, while no effect was observed in the mutated reporter (Figure 5(b)). Consistently, Western blot showed that miR-627-3p overexpression reduced the protein level of HMGA2 in Huh7-1.3 and HepG2.2.15 cells (Figure 5(c)). We further applied miR-627-3p inhibitor that reduced the level of miR-627-3p in HBV-HCC cells (Figure 5(d)). Transfection of miR-627-3p inhibitor in Huh7-1.3 and HepG2.2.15 cells increased protein level of HMGA2 in HBV-HCC cells (Figure 5(e)). Therefore, these data suggest miR-627-3p negatively regulates the expression of HMGA2.

### 3.6. HMMR-AS1 regulates the malignant phenotype of HBV-HCC cells through miR-627-3p/HMGA2 axis

**Figure 6.**
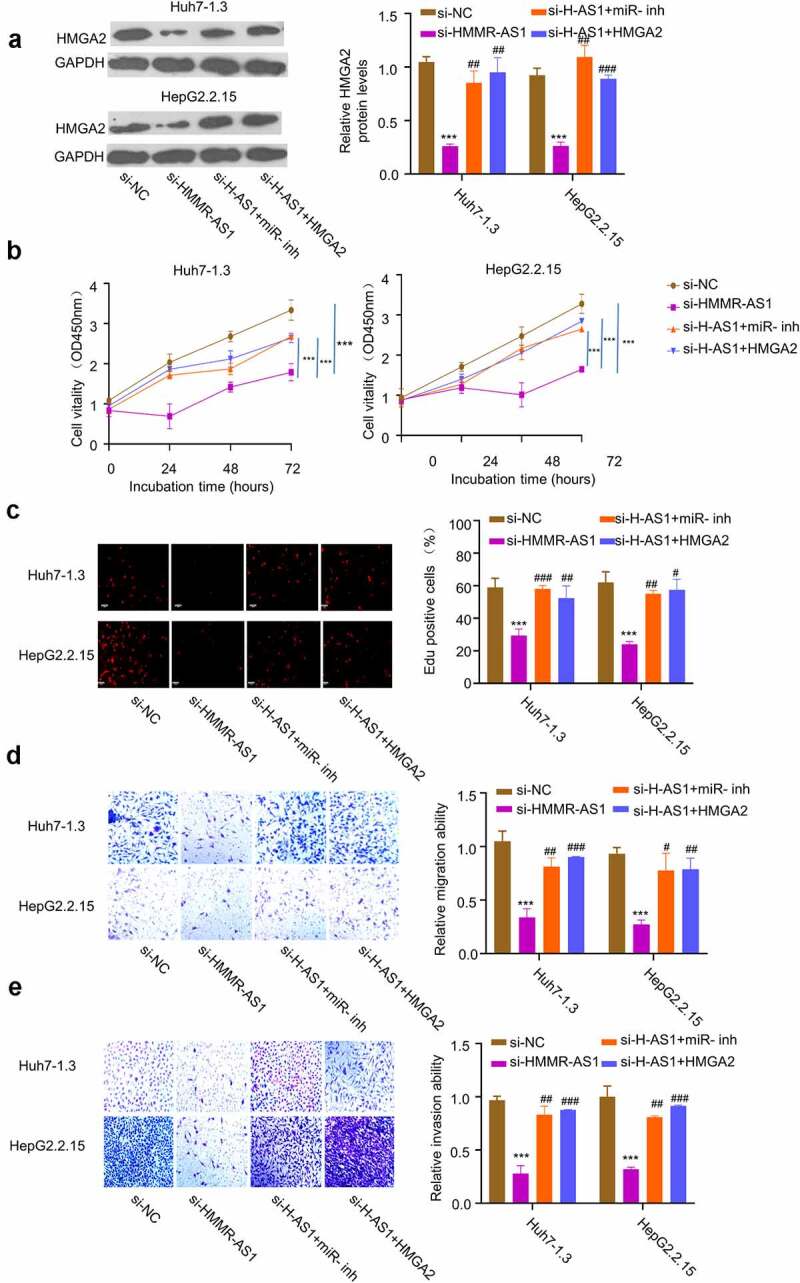
HMMR-AS1 regulates malignant behaviors of HBV-HCC cells via miR-627-3p/HMGA2 axis. Huh7-1.3 and HepG2.2.15 cells were transfected with si-HMMR-AS1 alone, or co-transfected with miR-627-3p inhibitor or HMGA2 expression vector. (a) HMGA2 protein levels were detected by Western blot. (b) and (c) Cell proliferation and DNA synthesis rate were measured by CCK8 and EdU assays. (d) and (e) Cell migration and invasion abilities of HBV-HCC cells were determined by wound-healing assay and transwell invasion assay.

To confirm the role of miR-627-3p/HMGA2 axis as the downstream regulators of HMMR-AS1, we transfected Huh7-1.3 and HepG2.2.15 cells with si-HMMR-AS1, and in the presence of miR-627-3p inhibitor or HMGA2 expression vector. The knockdown of HMMR-AS1 reduced the expression of HMGA2, while the co-transfection with miR-627-3p inhibitor or HMGA2 overexpression vector could partially rescue the protein level of HMGA2 (Figure 6(a)). miR-627-3p inhibitor or HMGA2 overexpression also rescued the inhibitory effects of HMMR-AS1 knockdown on cell proliferation (Figure 6(b)), EdU incorporation (Figure 6(c)), cell migration (Figure 6(d)), and cell invasion (Figure 6(e)). Overall, our data indicate that miR-627-3p/HMGA2 axis as the downstream regulators to mediate the effects of HMMR-AS1 on the malignant phenotype of HBV-HCC cells.

## 4. Discussion

Increasing evidence shows that non-coding regions of genes can be spliced into different lncRNAs, thereby affecting downstream gene expression [24]. LncRNAs play key roles in a variety of physiological and pathological conditions [25]. Among them, HMMR-AS1 is a recently discovered lncRNA, which is widely expressed in a variety of tumors, such as epithelial ovarian cancer, lung adenocarcinoma, radiosensitizes glioblastoma, and breast cancer [^20–23^]. Several studies have shown that HMMR-AS1 is implicated in tumor progression. Our data showed that HMMR-AS1 is upregulated in HBV-HCC tumor tissues and cells. The knockdown of HMMR-AS1 suppresses cell proliferation, invasion, and migration. Interestingly, HBV-encoded effector protein HBX seems to upregulate the expression of HMMR-AS1 in HBV-HCC. Future work is needed to delineate the mechanisms of how HBX regulates HMMR-AS1 expression.

It is widely reported that lncRNAs control tumor progression by affecting the expression of target miRNAs. miRNAs are a class of non-coding RNAs with 18–24 nucleotides [26]. They regulate physiological activities in cells by post-transcriptionally targeting mRNAs [27]. A previous study in lung cancer found that HMMR-AS1 can bind to miR-138 to regulate sirt6 expression and regulate the malignant progression of lung cancer [20]. In this study, we found that HMMR-AS1 targets miR-627-3p and negatively regulates its expression. miR-637-3p was reported to suppress tumor progression and invasion in osteosarcoma cells [15]. In addition, miR-627-3p could inhibit the expression of TGFB2 and the secretion of TGF-β, which leads to the downregulation of ZEB1 and the suppression of EMT in esophageal squamous cell carcinoma [18]. Our data suggest that miR-627-3p is an antagonistic mediator for the HMMR-AS1 in HCC.

We further demonstrated that miR-627-3p can target the 3’-UTR of HMGA2 mRNA and downregulate the expression of HMGA2 in HBV-HCC. Indeed, many studies have shown that HMGA2 plays an important role in the malignant process of HCC, and in addition to the miR-627-3p, there are multiple miRNAs, such as miR-497, miR-33a-5p, miR-196-5p, let-7b-5p implicated in the regulation of HMGA2 in different contexts [^28–31^]. HMGA2 can affect the infiltration of HepG2.2.15 cells as a downstream mediator of Lin28a/let-7a axis [32]. Interestingly, HBX has been reported to upregulate HMGA2 expression and enhance the proliferation, EMT, invasion and migration in HCC cells [33]. Consistently, we showed that HMGA2 overexpression can rescue the effect of HMMR-AS1 silencing on cell proliferation and invasion. Together our data suggest that HMMR-AS1 regulates miR-627-3p/HMGA2 axis to modulate the malignant phenotype of HCC cells.

## 5. Conclusion

In summary, we reported that HMMR-AS1 interacts with miR-626-3p and regulates its expressions in HBV-HCC cells. HMMR-AS1 expression level is negatively correlated with miR-626-3p expression. In addition, miR-627-3p binds to HMGA2 mRNA and negatively regulates HMGA2 expression in HBV-HCC cells. miR-627-3p/HMGA2 axis mediates the effects of HMMR-AS1 on the malignant phenotype of HBV-HCC cells. Future work is required to explore how HBX protein regulates HMMR-AS1/miR-627-3p/HMGA2 axis.

## Data Availability

The data is available from the corresponding author on reasonable request.
